# Field sales force model to increase adoption of a novel tuberculosis diagnostic test among private providers: evidence from India

**DOI:** 10.1136/bmjgh-2020-003600

**Published:** 2020-12-29

**Authors:** Sarang Deo, Pankaj Jindal, Manisha Sabharwal, Aparna Parulkar, Ritu Singh, Rigveda Kadam, Harkesh Dabas, Puneet Dewan

**Affiliations:** 1 Max Institute of Healthcare Management, Indian School of Business, Mohali, Punjab, India; 2 Operations Management, Indian School of Business, Hyderabad, Telangana, India; 3 Clinton Health Access Initiative, New Delhi, India; 4 Bill and Melinda Gates Foundation, New Delhi, India

**Keywords:** diagnostics and tools, health services research, health systems, public health, tuberculosis

## Abstract

**Background:**

Impact of novel high-quality tuberculosis (TB) tests such as Xpert MTB/RIF has been limited due to low uptake among private providers in high-burden countries including India. Our objective was to assess the impact of a demand generation intervention comprising field sales force on the uptake of high-quality TB tests by providers and its financial sustainability for private labs in the long run.

**Methods:**

We implemented a demand generation intervention across five Indian cities between October 2014 and June 2016 and compared the change in the quantity of Xpert cartridges ordered by labs in these cities from before (February 2013–September 2014) to after intervention (October 2014–December 2015) to corresponding change in labs in comparable non-intervention cities. We embedded this difference-in-differences estimate within a financial model to calculate the internal rate of return (IRR) if the labs were to invest in an Xpert machine with or without the demand generation intervention.

**Results:**

The intervention resulted in an estimated 60 additional Xpert cartridges ordered per lab-month in the intervention group, which yielded an estimated increase of 11 500 tests over the post-intervention period, at an additional cost of US$13.3–US$17.63 per test. Further, we found that investing in this intervention would increase the IRR from 4.8% to 5.5% for hospital labs but yield a negative IRR for standalone labs.

**Conclusions:**

Field sales force model can generate additional demand for Xpert at private labs, but additional strategies may be needed to ensure its financial sustainability.

**Key questions**

**What is already known?**

- Uptake of new diagnostic tests for tuberculosis such as Xpert MTB/RIF among private providers in high-burden countries is limited despite sufficient awareness.

**What are the new findings?**

- An intervention involving field sales force that made regular visits to private providers engaged with hospital and standalone labs increased the demand for Xpert.
- The demand generation intervention may yield positive financial returns on investment in Xpert for hospital labs but not for standalone labs.

**What do the new findings imply?**

- Alternate approaches such as digital marketing may be needed to improve the financial sustainability of demand generation activities.
- Wherever appropriate, public health system should explore contracting with private sector labs to efficiently use existing Xpert capacity and to expand access to testing.

## Background

Tuberculosis (TB) continues to be one of the biggest global health challenges of the 21st century. In 2018, more than 10 million individuals fell ill with TB and deaths due to TB were almost two times that of HIV.^1^ In the absence of an effective vaccine, the strategy for global fight against TB hinges on early and accurate diagnosis followed by high treatment compliance and success rate.^2^ A major technical breakthrough in this fight was the development of Xpert MTB/RIF (hereafter Xpert), the first rapid molecular test for diagnosing TB.^3^ Xpert is significantly more accurate than sputum smear microscopy, the mainstay of diagnostic algorithms in many national TB control programmes, and significantly faster than liquid culture test, the gold standard that is rarely used at scale.^4^ It was widely hailed by experts as a game changer^5–8^ and was included in the International Standards of TB Care by the WHO for microbiological diagnosis of all adults and children suspected of having pulmonary and extrapulmonary TB.^9^ However, growing evidence suggests that its actual impact may have fallen short due to limited uptake among private healthcare providers who diagnose and treat large fraction of patients with TB in high-burden countries.^10–12^

India, with an estimated 2.7 million TB cases, accounted for more than one-fourth of the estimated global incidence as well as deaths.^1 13^ The National Strategic Plan for 2017—2025 includes ambitious targets for the number of TB cases that are microbiologically confirmed, notified, initiated on treatment and offered drug-susceptibility testing.^14^ Achieving these targets requires rapid increase in the uptake of Xpert among private providers, who are estimated to treat more than 50% of the TB cases in India^15 16^ and are the most common first point of contact for patients.^17^ Accordingly, Xpert is included in the Standards of TB Care in India.^14 18^ However, adherence to these standards requires changing diagnostic practices of private providers, who commonly resort to empirical treatment using broad-spectrum antibiotics to rule out other conditions instead of high specificity microbiological tests for TB.^19 20^ They also order diagnostic tests that may lack scientific validity but provide attractive financial returns to themselves and private laboratories, distributors and manufacturers.^21 22^

Traditionally, private provider engagement strategies for TB control in India have not accounted for such incentives. Instead, they have relied on training and sensitisation of providers on appropriate diagnostic tests and algorithms, conducted by non-governmental organisations in partnership with the Revised National TB Control Program (now called National Tuberculosis Elimination Program).^23 24^ These efforts have met with limited success because private providers do seem to already possess the knowledge about standards of TB care but fail to translate this knowledge into appropriate behaviour due to financial and operational barriers, leading to a ‘know–do’ gap.^25–27^ Evidence from developed markets also suggests that closing the know–do gap and increasing the adoption of new medical technology by providers requires demand generation interventions that are targeted at individual physicians, such as detailing visits by sales personnel as against dissemination in large groups through didactic continuing medical education (CME) seminars.^28–30^ In low-income and middle-income countries, these methods have been used for social marketing of rapid diagnostic tests (RDTs) for malaria among private medical retailers, although with mixed results.^31 32^ Lessons from those interventions are unlikely to carry over seamlessly to the case of Xpert, which is much more expensive than RDTs, requires capital investment and needs to generate economic returns for private laboratories as well as private providers.

In this study, we report on one specific demand generation intervention, where field sales force regularly visited private providers with primary objective of bridging their ‘know–do’ gap about Xpert and thus increasing its uptake. First, we estimate the impact of this intervention on the number of additional Xpert tests ordered by private providers. Second, we combine this estimate with the costs of the intervention and other financial details to evaluate the internal rate of return to private laboratories from their investment in a GeneXpert machine (the four-module machine used to perform Xpert testing) to assess the financial sustainability of the intervention.

## Methods

### Study context

In 2013, a consortium (Initiative for Promoting Affordable and Quality TB Tests or IPAQT) of private laboratories (categorised as national chain, regional chain, standalone and hospital labs), along with the manufacturers and distributors, was formed. Its objective was to transform the market of the WHO-endorsed tests from high margin, low volume to low margin, high volume. Under this initiative, manufacturers and distributors agreed to provide test cartridges at reduced input costs to the member labs who, in turn, agreed to set the retail price of these tests at or below a predetermined ceiling price that yielded acceptable margins to all players across the value chain.^33^ A secretariat, funded by the Bill and Melinda Gates Foundation and staffed by the Clinton Health Access Initiative, designed and implemented the initiative (including the determination of the ceiling price) and undertook monitoring and evaluation efforts to ensure compliance by the consortium members.

**Figure 1:**
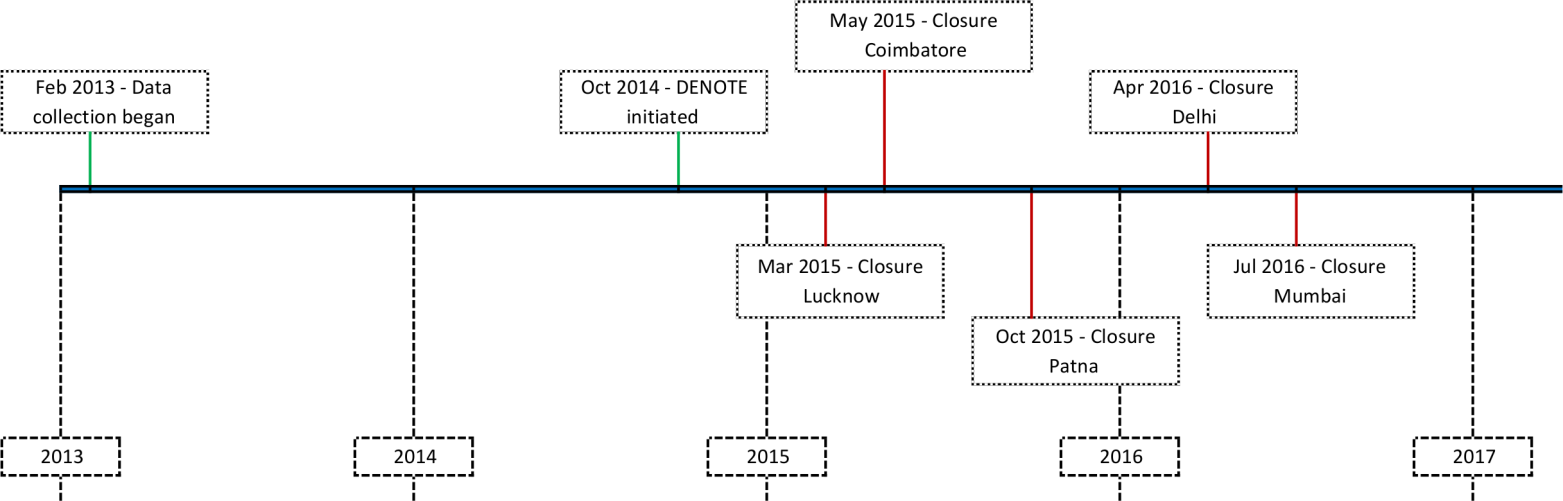
Timeline for IPAQT and DENOTE interventions. DENOTE, Demand Generation and Notification Effort.

Embedded within the overall initiative, IPAQT secretariat designed and implemented an intervention, termed as Demand Generation and Notification Effort (DENOTE), targeted at private healthcare providers in five Indian cities (New Delhi, Mumbai, Lucknow, Patna and Coimbatore) between October 2014 and June 2016 (figure 1). This intervention employed a sales force comprising 15 field representatives, supervised by area managers and a project manager. The field representatives and supervisors had prior experience in marketing pharmaceutical products to private providers and were provided initial training in the technical details of Xpert as well as the TB epidemic in India. A list of private providers for each city was prepared based on syndicated market research data (IMS Health) and was validated through an audit of prescriptions at the retail chemists. For cities of Mumbai and Patna, providers engaged in another large-scale private sector pilot were removed to avoid duplication of effort.^34^

The field representatives made routine visits to the private providers, where the visit frequency depended on the qualification and patient volume of the provider. General physicians (MBBS) with low patient volume (up to 10 patients per month) were visited once every 2 months, specialists (MD, Internal Medicine) with medium patient volume (10–20 patients per month) were visited once a month; whereas chest physicians (MD, pulmonology) with medium to high patient volume (more than 20 patients per month) were visited two times per month. In these visits, the field representatives provided information to the providers about the availability of these tests at affordable prices in a nearby IPAQT lab and reminded them about technical aspects of the tests. In addition, the field representatives also facilitated notification of TB cases from private labs and providers to a centralised government database. The personnel cost and the associated overheads for DENOTE were borne by the IPAQT secretariat. Concurrent to DENOTE, IPAQT conducted more than 40 CME seminars in partnership with local labs with average attendance of around 150 providers.

### Data

We used data on the quantity and timing of orders for Xpert cartridges placed by 79 IPAQT member laboratories with the exclusive distributor over 35 months comprising 20 months of pre-intervention (February 2013–September 2014) and 15 months of post-intervention (October 2014–December 2015) periods. In addition, we collated dates and locations of CME seminars conducted in the intervention and non-intervention cities over the study period. We excluded orders placed by national and regional chain laboratories because we did not have data on their internal allocation of the centrally procured Xpert cartridges among locations spanning intervention and non-intervention cities. We also excluded orders from four standalone and hospital laboratories that received samples from another large-scale private provider engagement pilot in Patna and Mumbai to avoid any confounding.^34^ Applying these exclusions yielded a panel dataset of 1785 lab-month observations (51 laboratories over a period of 35 months), of which 328 observations were zeros.

In addition, we obtained administrative data on the direct and indirect costs associated with the DENOTE intervention. Direct costs included salaries of managerial staff and field sales force involved in the intervention, whereas indirect costs included costs of shared corporate services such as information technology, accounting and finance.

Finally, we conducted semistructured interviews of senior managers of standalone and hospital labs to collect financial data (eg, revenues, fixed and variable costs) associated with installing and operating GeneXpert machine. We complemented it with operational data on the capacity of the equipment, its utilisation and error rate through discussions with technical experts in IPAQT.

### Analysis

To calculate the impact of DENOTE intervention on test orders, we conducted a difference-in-differences (DiD) analysis, where we compared the change in the quantity of Xpert cartridges ordered per month by labs in the intervention cities of New Delhi, Mumbai and Patna (intervention group) with the change in the quantity ordered by labs in 20 comparable non-intervention cities (control group, see online supplemental table 1). We excluded two intervention cities, Lucknow and Coimbatore, from the analysis due to the absence of standalone and hospital laboratories from these cities.

10.1136/bmjgh-2020-003600.supp1 Supplementary data

We ensured that the control and intervention cities had similar trends of the outcome variable (order quantity) before the intervention (online supplemental text 1). We fitted a set of linear panel data regression models whose main outcome variable was the number of Xpert cartridges ordered by a lab in a month. In the simplest (unadjusted) model we included only one binary predictor variable to indicate whether an observation belonged to the intervention or control group, before or after the start of the intervention. In subsequent (adjusted) models, we added a continuous time trend to capture any secular growth in Xpert orders by private providers, lab fixed-effects to account for time-invariant factors specific to individual laboratories (eg, type of laboratory, its network of collection centres, its management practices) and time fixed-effects to account for seasonality in the orders (eg, end of the financial year). In the fully adjusted model, we added more covariates to control for the effect of CME seminars conducted in the intervention as well as the control cities to generate awareness about Xpert among providers. These included a binary variable to indicate whether a CME seminar was organised in the city of a standalone or hospital laboratory in the previous month and a continuous variable denoting the number of CMEs conducted in the city of that laboratory until the current month. The former captured the effect of the most recent CME, whereas the latter captured the cumulative effect of all CMEs done until date on the prescription behaviour of private providers in the city (possibly through increased awareness). We used the estimated effect of the intervention per month per laboratory from the fully adjusted model to calculate the overall impact of the intervention, that is, the number of additional Xpert cartridges ordered in the intervention cities compared with non-intervention cities over the course of the DENOTE intervention.

10.1136/bmjgh-2020-003600.supp2 Supplementary data

We assessed the robustness of our results by estimating variants of the model that contained additional variables to account for potential leading and concurrent effects of CME seminars such as procurement of cartridges by labs during or before the month of CME in anticipation of increased Xpert demand in the following months. We performed all the above analyses using packages available in R.

Based on the discussions with senior managerial staff in the IPAQT secretariat, we apportioned two-thirds of the total cost of the DENOTE intervention to demand generation activities and the rest to notification efforts. We then allocated the demand generation cost among standalone and hospital laboratories using two methods: (1) based on the proportion of laboratories that belong to these categories in the intervention group, and (2) based on the proportion of Xpert orders contributed by these categories to all the Xpert cartridges ordered in the intervention group in 2015. We used these allocated costs and the estimate of the number of additional Xpert tests ordered in the intervention cities over the course of the DENOTE intervention to compute the cost per additional Xpert ordered.

To assess the financial sustainability of the DENOTE intervention under regular market conditions, we developed a financial model for standalone and hospital lab’s investment in a new GeneXpert machine for a time-horizon of 4 years under two scenarios. In the first scenario, we assumed that the demand generation activities were stopped and hence the labs did not incur the corresponding costs. However, they incurred annual fixed costs (eg, direct operating and maintenance expenses, marketing expenses, as well as allocation of indirect administrative and other lab overheads), as well as variable costs (eg, cost of the cartridge, consumables, specimen handling, channel margins). For calculating the revenues, we assumed the IPAQT negotiated retail price of 2000 Indian rupees per TB test, as well as commercial prices of 4500 Indian rupees and 3750 Indian rupees for HIV and hepatitis C virus (HCV) tests, respectively. We calculated the capacity of a four-module GeneXpert machine assuming cycle time of 2 hours, 12 operating hours per day and 26 operating hours per month. For the standalone lab, to reflect the lack of captive demand and gradual business growth, we assumed that the TB tests would contribute to 2% utilisation of the machine capacity in the first month of operation and reach a steady state of 50% utilisation over 4 years. For the hospital lab, which may have access to patient referrals from affiliated doctors, we assumed a constant 25% utilisation over the 4-year horizon. In addition, we assumed that the HIV and HCV tests together accounted for 10% utilisation of the machine capacity where the volume of the former test was five times that of the latter. In the second scenario, we assumed that the demand generation activities were conducted by the lab and hence they incurred the corresponding costs. To calculate these costs, we excluded the personnel cost of the IPAQT secretariat allocated to the DENOTE intervention but retained all other components and recalculated the cost per test, and subsequently the total cost associated with demand generation activities. We then added these to the other variable and fixed costs considered in the first scenario and calculated the total cost incurred by the labs. Similarly, to calculate the total revenue for this scenario, we considered the impact of these demand generation activities on the monthly volume of Xpert tests as estimated from our DiD analysis, and consequently on the revenue, and added these to the revenues considered in the first scenario. Using these inputs, we calculated the internal rate of return over 4 years for each of the scenarios and each type of lab.

### Patient and public involvement

We did not have any patient or public involvement in the design or conduct of this study.

## Results

Table 1 shows key descriptive statistics for the final dataset used in the analysis pertaining to labs’ ordering behaviour. Total order quantity in the intervention group increased by 17 080 cartridges, whereas that in the control group increased by 13 190 cartridges. This corresponded to an increase of 98.03 cartridges per lab-month in the intervention group compared with 26.39 cartridges per lab-month in the control group. The number of orders per lab-month increased by about 0.25 in both the groups. The combined effect of these is reflected in the order quantity per order, which increased by 103.70 cartridges per order for intervention group but only by 7.85 cartridges per order for the control group.

**Table 1:** Descriptive statistics of ordering behaviour of laboratories

	Intervention group			Control group		
	Before	After	Difference	Before	After	Difference
Total order quantity	8140	25 220	17 080	7420	20 610	13 190
Total no of orders	35	75	40	75	193	118
Order quantity per lab-month	31.31	129.33	98.03	9.76	36.16	26.39
No of orders per lab-month	0.13	0.38	0.25	0.10	0.34	0.24
Order quantity per order	232.57	336.27	103.70	98.93	106.79	7.85

Intervention group includes five hospital labs and eight standalone labs. Control group includes 24 hospital labs and 14 standalone labs. ‘Before’ period comprises 20 months from February 2013 to September 2014. ‘After’ period comprises 15 months from October 2014 to December 2015.

Table 2 shows the estimation results for the unadjusted and various adjusted versions of the DiD model. After including fixed effects for laboratories and month-of-year and controlling for a time trend and the effects of CMEs (column (4)), coefficient of DENOTE indicated that the intervention was associated with approximately 60 additional Xpert cartridges per month per laboratory on average (p value=0.023, 95% CI: 8.495 to 111.501). Aggregating this effect over 13 intervention laboratories and an intervention period of 15 months yielded an estimated total of 11 700 additional Xpert cartridges ordered. The coefficient for ‘Time’ indicated that every additional month was associated an additional 1.57 cartridges per lab-month (p value=0.005, 95% CI: 0.463 to 2.677), reflecting the secular growth in uptake of Xpert. Having conducted a CME seminar in the previous month was associated with 36 additional Xpert cartridges (p value=0.064, 95% CI: −2.077 to 73.319) but the cumulative number of CMEs conducted to date was not found to be statistically significant. The effect of DENOTE along with that of ‘Time’ and ‘CME in previous month’ predictor variables continued to be statistically significant in all three model variants considered for the robustness analysis (online supplemental table 2).

10.1136/bmjgh-2020-003600.supp3 Supplementary data

**Table 2:** Estimates of the difference-in-differences models

	(1)	(2)	(3)	(4)
DENOTE	106.585***	96.312***	69.017***	59.998**
	(78.643 to 134.527)	(65.639 to 126.984)	(35.300 to 102.735)	(8.495 to 111.501)
Time			1.658***	1.570***
			(0.792 to 2.524)	(0.463 to 2.677)
CME in previous month				35.621*
				(−2.077 to 73.319)
No of CMEs conducted				2.178
				(−13.869 to 18.224)
Month and lab fixed effects	N	Y	Y	Y
Observations	1785	1785	1785	1785
Adjusted R^2^	0.030	0.267	0.272	0.273

Each cell contains the coefficients and 95% CIs in parentheses obtained from a difference-in-differences model fitted using least squares method, where each observation is at the lab-month level. DENOTE refers to the presence of the intervention in a particular lab in a particular month. *P<0.10, **p<0.05, ***p<0.01.

CME, continuing medical education; DENOTE, Demand Generation and Notification Effort.

Table 3 shows that the total cost of implementing the DENOTE intervention during the study period was $466 805. Of this, around 88% was direct cost comprising the cost of personnel in the IPAQT secretariat and cost of staffing from the implementing agency, whereas the remaining 12% was indirect cost. Based on the expert opinion of senior management staff, two-thirds of this cost ($311 203) was attributed to demand generation activities across all labs.

**Table 3:** Summary of costs associated with DENOTE intervention

Cost component	Amount (US$)
Field staff cost*	258 310
Managerial staff cost†	141 071
Other direct cost‡	12 757
Total direct cost	412 138
Indirect cost§	54 667
Total cost for DENOTE	466 805
Cost attributed to demand generation¶	311 203

*Field staff cost includes salaries and benefits of field sales force and area manager.

†Managerial staff cost includes salaries and benefits of project manager and other members of the technical team including analysts.

‡Other direct cost includes travel, events and other incidental expenses directly attributable to the DENOTE intervention.

§Indirect cost includes shared services such as IT, finance and accounting.

¶Cost attributed to demand generation is 66% of the total cost of DENOTE intervention based on inputs from senior management staff.

DENOTE, Demand Generation and Notification Effort; IT, information technology.

Table 4 shows that $155 602–$206 276 of this amount was attributable to standalone and hospital labs based on the two allocation methods described earlier. Dividing this over 11 700 additional tests estimated to be the impact of demand generation activities (calculated from the regression estimates), translated to a unit cost of $13.3–$17.6 per test. These unit costs reduced to $7.7–$10.2 per test under the hypothetical scenario of the labs conducting demand generating activities. Inserting the average of these estimates in the financial model described earlier, we found that the hospital lab would earn an internal rate of return of 4.8% if the demand generation activities were stopped entirely and 5.5% if they undertook these activities for 1 additional year. The corresponding numbers for a standalone lab were −0.90% and −0.46%, respectively.

**Table 4:** Allocation of demand generation costs to hospital and standalone labs

	Method 1	Method 2
	(No of labs)	(No of cartridges)
Share of standalone and hospital labs*	50.0%	66.3%
Demand generation costs allocated to standalone labs and hospitals (US$)	155 602	206 276
Demand generation cost per test (US$)	13.30	17.63
Demand generation cost per test (INR)†	864	1146

*13 out of 26 labs (50%) in the intervention group are standalone and hospital labs; 37 800 of 57 028 cartridges ordered in the intervention group are by standalone and hospital labs.

†Approximate exchange rate during the intervention period was US$1=65 INR.

INR, Indian rupee.

## Discussion

The impact of Xpert on the global TB epidemic is constrained by limited uptake among private providers in high-burden countries. However, there is limited information about which strategies are likely to be effective in increasing this uptake in the private sector. In this study, we provided one of the first pieces of quantitative evidence regarding the impact of a demand generation initiative involving a field sales force on the uptake of Xpert among private labs and providers in India. We found that the intervention was effective, that is, it generated incremental uptake of Xpert over and above the status quo and conventional provider sensitisation methods such as CME seminars, which were found to have additive demand generation impact of their own, as evidenced by the consistent significant effect of the variable ‘CME in previous month’ in all model variants. We also found partial support for the field sales force intervention to be financially sustainable. Although the unit cost of the demand generation activities per additional test ordered was comparable to the procurement cost of the test cartridges, results of our financial modelling analysis showed that incurring this additional cost increased the internal rate of return on investment in Xpert machines for private labs.

Most of the existing evidence about usage of Xpert comes from multicentric trials in Latin America and sub-Saharan Africa.^35–37^ However, those studies were based in the public health systems of the respective countries and did not involve any demand generation activities aimed to improve Xpert uptake by providers. An education and outreach programme covering more than 3500 public and private providers in four Indian cities more than doubled the paediatric TB case detection rate.^38^ In that project Xpert was available to patients free of cost and the testing was conducted in a dedicated high through-put laboratory. In contrast, our project was based on the market dynamics between private labs and providers and required patients to pay out-of-pocket for the test reflecting the reality of India’s private health sector. A detailed costing of that intervention found a substantially lower outreach cost ($0.6–$2.5 per referred patient) compared with our intervention.^39^ However, this is likely attributable to a lower intensity of the outreach activities and a narrow set of target providers. In that study, project team made over 2200 one-on-one visits to 3670 providers over a period of 27 months, whereas the field sales force in our intervention made 0.5–2 visits per provider per month. Furthermore, the study did not report incremental impact of the intervention over a set of non-intervention providers thereby making direct comparison of the cost-effectiveness of two approaches difficult.

Several interventions have evaluated the effectiveness of social marketing approaches to increase the uptake of RDTs for malaria across low-income countries in Africa and Asia.^32^ These interventions differ from ours in important aspects due, in part, to the difference in characteristics of the test and the accompanying health systems. Most of these interventions targeted private medical retailers, who are the first point of contact for febrile patients in these settings.^40 41^ The tests are often available for free or highly subsidised prices to the patients and are supplied through a parallel distribution channel to the retailers at heavily subsidised prices.^42^ Most of these studies did not focus on comprehensive financial sustainability analysis, perhaps, because the retailers do not have to make substantial investments for RDTs unlike an Xpert machine. Furthermore, financial sustainability in our context was further complicated by the involvement of two self-interested, private players—labs and providers—whose efforts and economic incentives needed to be coordinated for the intervention to be successful. Consequently, the demand generation activity in our intervention focused on the providers and labs and relied more heavily on a field sales force, whereas that in the studies on RDTs involve mass media campaigns aimed at changing the behaviour of patients and community members.

The long-term scalability and sustainability of the demand generation intervention without external donor funding depends on whether it can generate substantial incremental financial return for private laboratories. Our analysis showed that private labs could earn a slightly higher financial return by investing in marketing activities than not investing after the donor support is discontinued. However, this return may still be lower than that obtained from competing avenues of investment (beyond Xpert and other TB tests). Hence, alternate mechanisms to improve the financial return should be explored. Increasing the ceiling price to achieve an acceptable internal rate of return may not be feasible as most patients pay out-of-pocket and increasing the price may reduce patient compliance with provider prescription of Xpert. Instead, private labs may find it financially more attractive to use the field sales force to promote a wider assortment of tests beyond TB, which can be facilitated by the expansion of GeneXpert machine for other molecular tests such as HIV and HCV viral load.^10^ The use of digital marketing campaigns and digital platforms presents another approach to promote the WHO-endorsed tests. However, despite seemingly being less costly, the effectiveness of this approach depends on the level of engagement of providers with the digital platform, which may require customisation of the content based on the learning styles of individual providers.^43^ Finally, in the case of some labs with low demand, the financial return on investment may be more attractive for one-module or two-module Xpert machines as those can achieve higher utilisation and incur a lower investment compared with four-module machines considered in our analysis.

We also found that the financial sustainability is substantially lower for standalone labs as compared with hospital labs. This is largely driven by our differential assumptions about testing volume at these two types of labs. Hospital labs are likely to get a large volume of captive patients from affiliated providers, whereas standalone labs will take time to gradually increase their testing volume. It is also widely known that they have to pay channel margins to referring providers,^22^ which may adversely impact return on their own investment. An important policy implication of our findings is that if private labs may stop investing in GeneXpert machines if they cannot improve their financial returns using above approaches. Given that a segment of presumptive patients with TB will continue to visit private providers, the National Tuberculosis Elimination Program may have to explore alternate models of expanding coverage of Xpert among them. These include either encouraging private providers to refer patients to government labs for testing or paying for Xpert tests conducted in private labs depending on available capacities and comparison of relevant costs in public versus private.

Our results should be interpreted with caution in light of the limitations of our analysis. We may have underestimated the impact of the marketing outreach activity as we did not include data from national and regional chain laboratories in our analysis. We partially mitigated this limitation in our cost-effectiveness analysis through appropriate allocation of total programmatic cost. We did not directly observe the increase in test prescriptions by providers in the intervention cities. However, orders placed by labs with distributors (source of our data) should roughly equal downstream demand, that is, the number of tests prescribed by providers over the study period of 35 months. Hence, we decided to conduct the analysis at the level of cities rather than individual providers. This aggregated measurement is also more appropriate for possible market-level impact of intervention, for example, providers not visited by the field sales force may also increase adoption of Xpert to compete effectively. To the best of our knowledge, cities in the control arm did not have any other significant private sector engagement intervention during our study period beyond the regular Public–Private Mix activities of the Revised National TB Control Program, which were also conducted in the intervention cities. On a related note, the intervention was implemented under the aegis of IPAQT and labs in both intervention and control arms were members of the IPAQT consortium. Hence, strictly speaking, our results may not be generalisable to other private labs, who may have found joining the consortium to be financially unattractive. Finally, we could not measure the impact of increased uptake of Xpert on patient outcomes. Previous pragmatic trials found significant increase in bacteriologically confirmed cases in the treatment arm (with Xpert) compared with the control arm (with smear microscopy) but not on overall case notification and mortality, perhaps due to replacement of empirical treatment with Xpert.^44 45^ This effect is likely to exist in our setting, where private providers are known to rely heavily on clinical diagnosis and empirical treatment initiation.^20 46^ Future research efforts could focus on conducting cost-effectiveness analysis that combine the health impact of these factors with the cost estimates from our analysis.

## Conclusions

In this study, we found that a demand generation intervention involving a field sales force that made routine visits to private providers was effective in increasing their adoption of Xpert reflected in the increased quantity of Xpert cartridges ordered by the private labs. The long-term financial sustainability of this intervention at the estimated level of effectiveness was not unequivocal and was higher for labs associated with a hospital compared with standalone labs. Further research is needed on improving the financial viability by adding more tests under the intervention and employing low-cost, high-reach digital marketing channels and assessing the cost-effectiveness of such an intervention in improving patient outcomes.
