# Supplementary material for: Field sales force model to increase adoption of a novel tuberculosis diagnostic test among private providers: evidence from India
Source: BMJ Glob Health. 2020 Dec 29;5(12):e003600. doi: 10.1136/bmjgh-2020-003600 (PMC7778745; doi:10.1136/bmjgh-2020-003600)
Supplement: Supplementary data [file bmjgh-2020-003600supp001.pdf]

Table 1: Details of the control group

**City**	**Hospital Labs**	**Stand-alone Labs**
Ahmedabad	1	2
Bangalore	4	
Bathalapalli	1	
Bhopal		1
Chandigarh		1
Chennai	3	1
Dharamshala	2	
Hisar		1
Hyderabad	1	1
Indore	1	
Kanpur		1
Kochi	2	
Kolkata	2	1
Mangalore	3	
Moradabad		1
Pune	2	2
Purnia		1
Raxaul	1	
Roorkee		1
Trivandrum	1	
**Grand Total**	**24**	**14**
