# Supplementary material for: Field sales force model to increase adoption of a novel tuberculosis diagnostic test among private providers: evidence from India
Source: BMJ Glob Health. 2020 Dec 29;5(12):e003600. doi: 10.1136/bmjgh-2020-003600 (PMC7778745; doi:10.1136/bmjgh-2020-003600)
Supplement: Supplementary data [file bmjgh-2020-003600supp002.pdf]

Checking for parallel trends assumption for Difference-in-Differences analysis

We estimated two models to check the parallel trends assumption on the data before the intervention.

In Model 1, we included a continuous variable for time and interacted it with a categorical variable to represent control vs. intervention arm. The coefficient of this interaction term was not significant (Column (1) in Figure 1 and left panel in Figure 2).

In Model 2, we included categorical variables representing months and interacted these with the categorical variable representing control vs. intervention arm. About half of these interaction variables were not significant (Column (2) in Figure 1 and right panel in Figure 2).

Model 1:

$V_{it} = \gamma_{lab} + \lambda_{moy} + \beta\ (t) + \delta\ (d\_t) + \varepsilon_{it}$,

where

i = index for labs

t = time index (goes from 1 to 20)

γ_{lab} = Lab fixed effects

λ_{moy} = Month-of-year fixed effect

d = whether i is denote-lab or not (1 if it is)

d_t = interaction term between d and t

Model 2:

$V_{it} = \gamma_{lab} + \lambda_{moy} + \beta_t + \delta_{d\_t} + \varepsilon_{it}$

where

β_t = time fixed effects (19 fixed effects)

δ_{d_t} = fixed effects for d_t (20 fixed effects because d_t would take values from 0 to 20)

Estimates of models to check the parallel-trends assumption

	Dependent variable:	
	Lab-month Volumes	
	Linear Time Trend (1)	Time Fixed Effects (2)
Time	1.20* (-0.04, 2.44)	
IsDenoteLab*Time	1.87 (-0.46, 4.20)	
FE Time 2		1.32 (-40.98, 43.61)
FE Time 3		1.05 (-41.24, 43.35)
FE Time 4		3.16 (-39.14, 45.45)
FE Time 5		2.63 (-39.66, 44.93)
FE Time 6		0.79 (-41.51, 43.09)
FE Time 7		0.79 (-41.51, 43.09)
FE Time 8		7.37 (-34.93, 49.66)
FE Time 9		8.95 (-33.35, 51.24)
FE Time 10		20.26 (-22.03, 62.56)
FE Time 11		7.37 (-34.93, 49.66)
FE Time 12		3.95 (-38.35, 46.24)
FE Time 13		8.95 (-33.35, 51.24)
FE Time 14		17.63 (-24.66, 59.93)
FE Time 15		14.47 (-27.82, 56.77)
FE Time 16		15.00 (-27.30, 57.30)
FE Time 17		28.95 (-13.35, 71.24)
FE Time 18		5.26 (-37.03, 47.56)
FE Time 19		24.74 (-17.56, 67.03)
FE Time 20		22.63 (-19.66, 64.93)
FE IsDenoteLab*Time 1		-63.39 (-145.44, 18.66)
FE IsDenoteLab*Time 2		-70.86* (-152.91, 11.19)
FE IsDenoteLab*Time 3		-62.90 (-144.96, 19.15)
FE IsDenoteLab*Time 4		-45.01 (-127.06, 37.04)
FE IsDenoteLab*Time 5		-69.10* (-151.15, 12.95)
FE IsDenoteLab*Time 6		-70.33* (-152.39, 11.72)
FE IsDenoteLab*Time 7		-59.56 (-141.62, 22.49)
FE IsDenoteLab*Time 8		-76.91* (-158.97, 5.14)
FE IsDenoteLab*Time 9		-78.49* (-160.55, 3.56)
FE IsDenoteLab*Time 10		-88.27** (-170.32, -6.22)
FE IsDenoteLab*Time 11		-15.37 (-97.43, 66.68)
FE IsDenoteLab*Time 12		98.82** (16.76, 180.87)
FE IsDenoteLab*Time 13		-78.49* (-160.55, 3.56)
FE IsDenoteLab*Time 14		-61.02 (-143.08, 21.03)
FE IsDenoteLab*Time 15		-26.33 (-108.38, 55.73)
FE IsDenoteLab*Time 16		-23.01 (-105.06, 59.05)
FE IsDenoteLab*Time 17		-67.72 (-149.78, 14.33)
FE IsDenoteLab*Time 18		69.04* (-13.01, 151.09)
FE IsDenoteLab*Time 19		-78.90* (-160.95, 3.16)
FE IsDenoteLab*Time 20		-92.18** (-174.23, -10.12)
Constant	64.47** (13.41, 115.53)	38.24 (-12.25, 88.73)
Lab Fixed Effects	Yes	Yes
Month-of-year Fixed Effects	Yes	No
Observations	1,020	1,020
R^2	0.21	0.25
Adjusted R^2	0.16	0.18
Note:		*p<0.1; **p<0.05; ***p<0.01

Figure 1: Estimation Results of Models 1 and 2

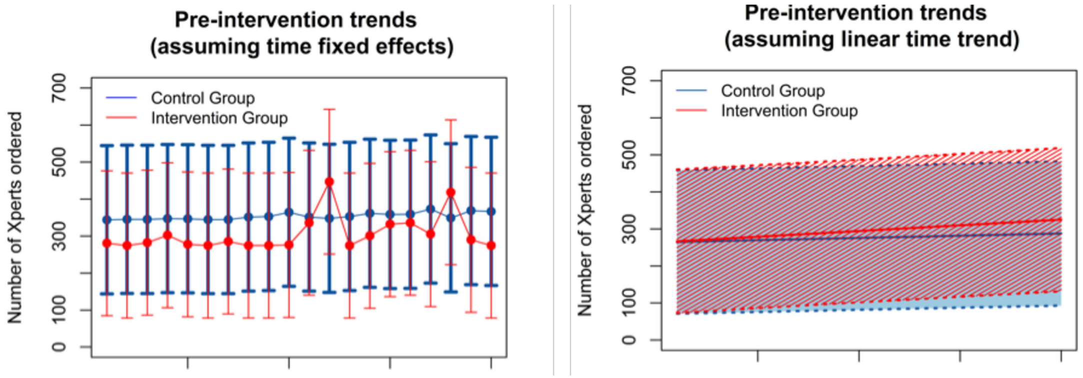

Figure 2: Pictorial representation of results of Models 1 and 2
