# Supplementary material for: Field sales force model to increase adoption of a novel tuberculosis diagnostic test among private providers: evidence from India
Source: BMJ Glob Health. 2020 Dec 29;5(12):e003600. doi: 10.1136/bmjgh-2020-003600 (PMC7778745; doi:10.1136/bmjgh-2020-003600)
Supplement: Supplementary data [file bmjgh-2020-003600supp003.pdf]

Estimates of the Difference-in-Differences model variants that control for contemporaneous and lead effects of CMEs

	(5)	(6)	(7)
DENOTE	54.509** (2.689, 106.329)	55.374** (3.458, 107.290)	49.090* (-3.193, 101.373)
Time	1.423** (0.305, 2.541)	1.476*** (0.362, 2.591)	1.310** (0.183, 2.438)
CME in previous month	34.500* (-3.194, 72.194)	36.444* (-1.263, 74.151)	35.338* (-2.358, 73.034)
CME in this month	-33.049* (-69.257, 3.159)		-35.051* (-71.340, 1.238)
CME in next month		24.758 (-10.773, 60.289)	27.237 (-8.359, 62.834)
No. of CMEs done	5.662 (-10.822, 22.146)	3.975 (-12.274, 20.223)	7.850 (-8.875, 24.574)
Month and Lab fixed effects	Y	Y	Y
Observations	1785	1785	1785
Adjusted R^2	0.274	0.273	0.274

Notes: Each cell contains the coefficients and 95% confidence intervals in parentheses obtained from a difference-in-differences model fitted using least squares method, where each observation is at the lab-month level. DENOTE refers to the presence of the intervention in a particular lab in a particular month. *p<0.10, **p<0.05, ***p<0.01
